# Basic knowledge in child protection– evaluation of an online-course for webbased transfer of interprofessional basic knowledge in child protection

**DOI:** 10.1192/j.eurpsy.2022.1135

**Published:** 2022-09-01

**Authors:** J. Bittner, A. Maier, J.M. Fegert, M. Rassenhofer, U. Hoffmann

**Affiliations:** Universityhospital Ulm, Child And Adolescent Psychiatry/psychotherapy, Ulm, Germany

**Keywords:** child protection, sexual violence against children, Child abuse

## Abstract

**Introduction:**

Insufficient or faulty cooperation between the various child protection professions can have an extremly negative impact on the well-being of the concerned children. Communication problems that were revealed when dealing with cases of child abuse show the importance of adequate cooperation and common language of the involved professions in child protection.

**Objectives:**

An online-course adressing medical-therapeutic professionals, youth welfare as well as judiciary and police was developed to impart skills and knowledge in child protection to create interdisciplinary understanding and improve cooperation between the involved professions.

**Methods:**

The acquisition of competencies, the transfer of learning content into everyday work and the quality of the online-course are determined using an online-survey before starting (t1) and after completing (t2) the course. T1-assessment is currently being evaluated with 1034 datasets, t2-assessment will take place 03/2022.

**Results:**

Intended target groups could be accessed and participated in the online-course, although the ratio of medical-therapeutic participants was greater than of judiciary professionals. Specific results of T1- and T2- assesssment and comparing analyses are expected in March 2022 and will be presented.

**Conclusions:**

Based on existing online-courses developed by the Universityhospital Ulm, the suitability of online-education for training professionals in the field of child protection could be proven. If comparable effects can be shown for this online-course, there is an increase in evaluated offers of high quality. These enable comprehensive and low-threshold access to the subject of interdisciplinary communication and cooperation in child protection for involved professionals.

**Disclosure:**

No significant relationships.

